# Actomyosin Activity and Piezo1 Activity Synergistically Drive Urinary System Fibroblast Activation

**DOI:** 10.1002/advs.202303369

**Published:** 2023-10-22

**Authors:** Guo Chen, Xiaoshuai Gao, Jiawei Chen, Liao Peng, Shuang Chen, Cai Tang, Yi Dai, Qiang Wei, Deyi Luo

**Affiliations:** ^1^ Department of Urology Institute of Urology (Laboratory of Reconstructive Urology) West China Hospital Sichuan University Chengdu Sichuan 610041 P. R. China; ^2^ Department of Urology and Pelvic surgery West China School of Public Health and West China Fourth Hospital Sichuan University Chengdu Sichuan 610041 P. R. China; ^3^ College of Polymer Science and Engineering State Key Laboratory of Polymer Materials and Engineering Sichuan University Chengdu Sichuan 610065 P. R. China

**Keywords:** actomyosin, fibroblasts, fibrosis, Piezo1, urinary Systems

## Abstract

Mechanical cues play a crucial role in activating myofibroblasts from quiescent fibroblasts during fibrosis, and the stiffness of the extracellular matrix is of significant importance in this process. While intracellular force mediated by myosin II and calcium influx regulated by Piezo1 are the primary mechanisms by which cells sense and respond to mechanical forces, their intercellular mechanical interaction remains to be elucidated. Here, hydrogels with tunable substrate are used to systematically investigate the crosstalk of myosin II and Piezo1 in fibroblast to myofibroblast transition (FMT). The findings reveal that the two distinct signaling pathways are integrated to convert mechanical stiffness signals into biochemical signals during bladder‐specific FMT. Moreover, it is demonstrated that the crosstalk between myosin II and Piezo1 sensing mechanisms synergistically establishes a sustained feed‐forward loop that contributes to chromatin remodeling, induces the expression of downstream target genes, and ultimately exacerbates FMT, in which the intracellular force activates Piezo1 by PI3K/PIP3 pathway‐mediated membrane tension and the Piezo1‐regulated calcium influx enhances intracellular force by the classical FAK/RhoA/ROCK pathway. Finally, the multifunctional Piezo1 in the complex feedback circuit of FMT drives to further identify that targeting Piezo1 as a therapeutic option for ameliorating bladder fibrosis and dysfunction.

## Graphical abstract

Mechanotransduction Circuit in Fibroblasts Responding to Stiffness. Myosin II and Piezo1 have a feed‐forward crosstalk through the PI3K/PIP3 pathway‐mediated membrane tension and the FAK/RhoA/ROCK pathway, which synergistically contribute to stiffness‐induced fibroblast activation. Additionally, Piezo1's crucial role has been further demonstrated in a mouse bladder fibrosis model, suggesting its potential as a novel therapeutic strategy for ameliorating bladder remodeling.

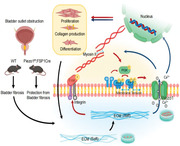

## 1 Introduction

Fibrosis is a pathological process characterized by the accumulation of excessive extracellular matrix (ECM), which can cause dysfunction or even failure of numerous organs and account for up to 50% deaths worldwide.^[^ ^1^, ^2^ ^]^ Bladder fibrosis, the common lower urinary fibrosis, is a hallmark of progressive partial bladder outlet obstruction (BOO), particularly in response to conditions such as chronic obstructive injury and benign prostatic hyperplasia (BPH).^[^ ^3^, ^4^ ^]^ There are over 80% men over 70 years old suffer from BPH, and the majority of them experience voiding and/or storage symptoms.^[^ ^5^, ^6^ ^]^ Regrettably, even after surgery to address the obstruction, many patients still experience lower urinary tract symptoms due to the irreversible pathological remodeling of the bladder wall.

During fibrogenesis, activated fibroblasts, also known as myofibroblasts, were the crucial mediators, which could secret multiple cytokines and chemokines to recruit immune cells and active neighboring quiescent fibroblasts.^[^ ^7^, ^8^ ^]^ To date, numerous strategies targeting such biochemical cues and related signaling pathways have been developed to reduce or even reverse bladder fibrosis,^[^ ^9^, ^10^ ^]^ yet few have yielded satisfactory results, possibly due to limited biophysical cues have been unmasked.

Emerging evidence has revealed various mechanical cues, like ECM stiffness or topography, can be transmitted as intracellular biomolecular signals to regulate the expression of target gene to mediate cellular behaviors.^[^ ^11^, ^12^, ^13^ ^]^ For sensing and transduction of mechanical cues, stretch‐activated ion channels and components of adhesion complexes have been proposed the major classes of mechanosensors.^[^ ^14^, ^15^ ^]^ Adhesion complexes consist of integrins, which interact with myosin to regulate various cellular processes.^[^ ^16^ ^]^ Myosin 2 (NMIIA) serves as the dominant generator of contractile forces in all non‐muscle cells and is intimately involved in numerous mechanotransduction pathways by interacting with actin filaments, thereby affecting the adhesion complexes.^[^ ^17^ ^]^ Alternatively, like many mechanically sensitive ion channels, Piezo1 involved in various cellular behavior by allowing calcium influx in response to different types of external forces.^[^ ^18^, ^19^, ^20^ ^]^ However, the independent function and interaction between these two mechanisms of mechanical cues sense and transduction in fibrosis remains unclear.

To address these questions, we systematically investigated the crosstalk of myosin II‐based intracellular force and Piezo1‐mediated calcium influx response to mechanical cues in bladder fibrosis. And we revealed that these two mechanosensors have a feed‐forward crosstalk by phosphatidylinositol 3‐kinase/phosphatidylinositol 3,4,5‐trisphosphate (PI3K/PIP3) pathway‐mediated membrane tension pathway and focal adhesion kinase (FAK)/RhoA/ROCK pathway. Above all, we provide evidence for Piezo1‐ and myosin II‐mediated pathways synergistically contribute to FMT. Finally, given the multifunctional Piezo1 in the complex feedback circuit of FMT, Piezo1 is demonstrated crucial during the mice bladder fibrosis model, which may likely offer a novel therapeutic strategy for ameliorating bladder remodeling.

## 2 Results

### 2.1 Bladder Fibroblasts are the Predominant Cell Type Involved in ECM Remodeling

**Figure 1:**
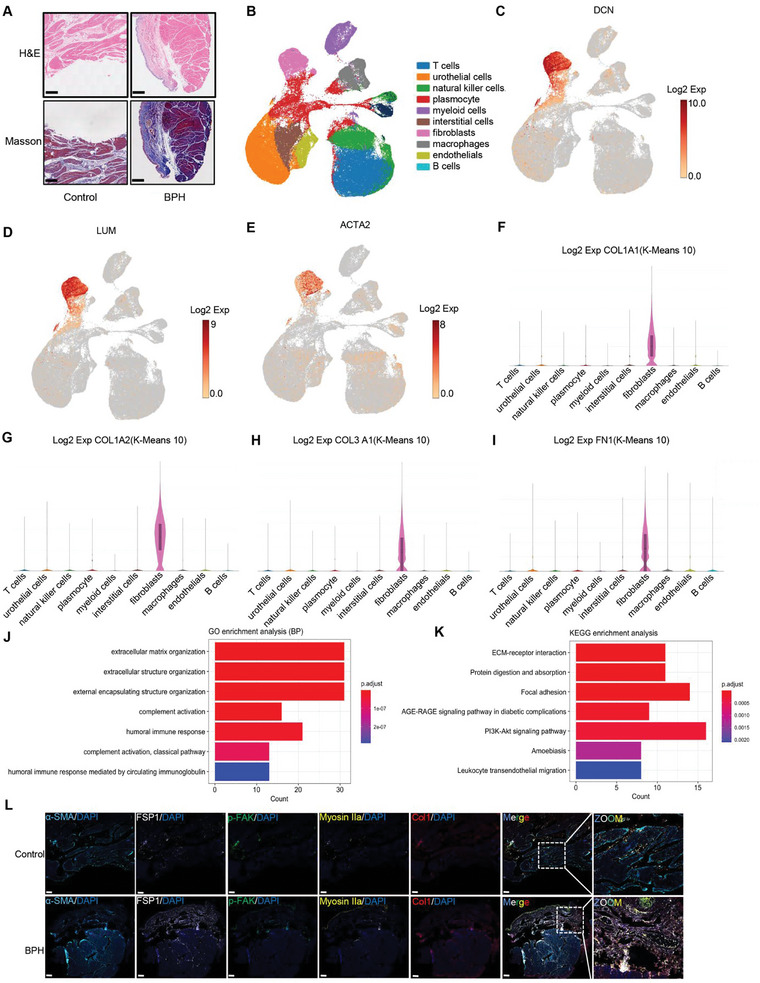
Bladder fibroblasts are the predominant cell type involved in ECM remodeling. A) Images of HE and Masson staining in BPH bladder and control bladder. Scale bar = 500 µm. B) Single‐cell of human bladders: Cell clusters found therein representing ten cell types (*n* = 5). C) Fibroblasts identified using canonical marker DCN. D) Fibroblasts identified using canonical marker LUM. E) Fibroblasts identified using canonical marker ACTA2. F) Expression of COL1A1 in each of the ten cell types, with the highest expression observed in fibroblasts. G) Expression of COL1A2 in each of the ten cell types, with the highest expression observed in fibroblasts. H) Expression of COL3A1 in each of the ten cell types, with the highest expression observed in fibroblasts. I) Expression of FN1 in each of the ten cell types, with the highest expression observed in fibroblasts. J) GO enrichment analysis of DEGs between BPH bladder and control bladder, showing that DEGs of fibroblasts were primarily involved in extracellular matrix remodeling. K) KEGG enrichment analysis of DEGs between BPH bladder and control bladder. L) Immunofluorescence was performed to co‐localize cellular traction force‐related markers and fibroblast activation markers (*n* = 3). Scale bar = 200 µm. Shown is the mean ± SD.

Bladder dysfunction caused by human BPH is mainly due to an increase in bladder collagens, as demonstrated by Masson and HE staining images (**Figure** **1**A). scRNA‐Seq was applied to investigate the cell subsets and their molecular functions in ECM deposition. After data normalization and quality control, ten cell types (Figure 1B) were identified using canonical marker genes (Table S3, Supporting Information). Fibroblasts and their activation states were marked by actin alpha 2 (ACTA2), lumican (LUM), and decorin (DCN) (Figure 1C–E), which predominantly expressed bladder collagens, including Collagen1 (Col1)a1, Col1a2, Col3a1, and Fibronectin (FN)1 (Figure 1F–I). Furthermore, Gene ontology (GO) enrichment analysis showed that deferentially expressed genes (DEGs) of fibroblasts were primarily involved in extracellular matrix remodeling (Figure 1J).

### 2.2 Bladder FMT might be Positively Related to Tissue Stiffness

The Kyoto Encyclopedia of Genes and Genomes (KEGG) enrichment analysis of DEGs in fibroblasts revealed enrichment in the focal adhesion and PI3K‐AKT signaling pathway, which are primarily associated with intracellular force transduction (Figure 1K). The DEGs enrichment analysis revealed an increase in canonical cellular force‐related markers such as FAK(*PTK2*), NMIIA(*MYH9*), ROCK(*ROCK*), etc, in BPH bladder compared to normal bladder (Table S4, Supporting Information). Additionally, the BPH bladder wall exhibited a significantly higher elasticity modulus of ≈32 kPa, while the normal bladder had an approximate modulus of 0.5 kPa (Figure S1, Supporting Information). To further explore the interplay between cellular traction force and BPH‐induced fibrotic bladder, immunofluorescence was performed to co‐localize cellular traction force‐related markers such as p‐FAK and p‐NMIIA with the fibroblast activation markers such as alpha smooth muscle Actin (α‐SMA) and Col1 in fibroblast marked by FSP1, and this observation led us to consider a positive correlation between cellular traction force and FMT in BPH bladder development (Figure 1L).

### 2.3 BOO‐Increased Bladder Wall Stiffness Leads to Bladder Remodeling in Mice

**Figure 2:**
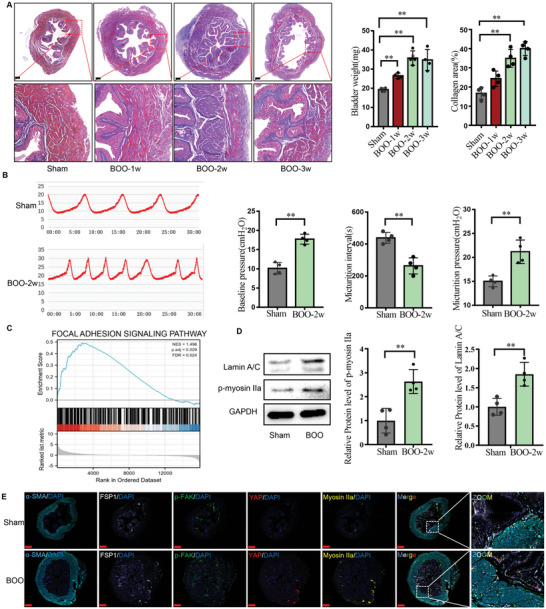
Cellular traction force and FMT were positively correlated in mice BOO bladder development. A) Masson of bladder tissues in sham‐operated group and BOO group; Scale bar = 200 µm. Bladder weight and collagen ration of sham‐operated group and BOO group (*n* = 4). B) Urodynamic curve(left) and quantification(right) of micturition intervals, baseline pressure, and micturition pressure of sham‐operated group and BOO group (*n* = 4). C) GSEA data showed that focal adhesion pathway significantly increased in BOO bladder. D) WB (left) and quantification(right) of p‐NMIIA and Lamin A/C in sham‐operated group and BOO group. E) Cellular traction force‐related markers are positively correlated with BOO‐induced FMT (*n* = 3); Scale bar = 500 µm.*p < 0.05, **p < 0.01; Shown is the mean ± SD.

Furthermore, male mice BOO models were established by externally ligating a 0.5 cm PE tube to the urethral root to investigate the potential correlation between intracellular traction force and fibrotic bladder development in mice BOO. After 1, 2, and 3 weeks of bladder obstruction, bladders were harvested and analyzed. Images of H&E and Masson staining showed a pronounced increase in collagen deposition and bladder weight after 2 weeks of obstruction (**Figure** **2**A). Additionally, urodynamic curve analysis of 2‐week obstruction showed that the micturition interval in the BOO group was significantly shorter than that in the sham‐operated group. Micturition pressure and baseline pressure were higher in the BOO group compared to the sham‐operated group (Figure 2B). These observations indicated that 2‐week obstruction instigates the obvious fibrosis collagen deposition and BOO dysfunction, making it a potentially appropriate time point for treatment.

Gene set enrichment analysis (GSEA) of focal adhesion pathway‐related genes revealed the pathway was activated in the mice BOO bladder (Figure 2C), suggesting the critical role of mechanotransduction in the BOO process. Microarray analysis of cell traction force‐related genes indicated that markers such as PI3K, Rac, etc. were significantly increased in BOO bladder (Figure S2A, Supporting Information), similar to what was observed in BPH bladder specimens. Additionally, the upregulation of bladder wall stiffness induced by BOO was observed through nanoindentation (Figure S2B, Supporting Information), which was further confirmed by the upregulated expression of the mechanical markers p‐NMIIA and Lamin A/C in bladder fibrosis (Figure 2D).This is consistent with the changes in bladder stiffness seen in human pathological BPH. To gain insights into the plausible involvement of cellular traction force in bladder FMT, we utilized multiple staining to co‐localize mechanical force markers including p‐FAK, p‐NMIIA, and p‐YAP with fibroblast activation markers including α‐SMA and Col1, in fibroblasts. The observations showed an increase in cellular traction force‐related markers positively correlated with fibroblast activation in BOO bladder (Figure 2E). These findings suggest a potential involvement of cellular traction force in bladder FMT in both human BPH bladder and mice BOO bladder.

### 2.4 Stiffness Regulates Bladder FMT via NMIIA‐Mediated Cellular Traction Force

**Figure 3:**
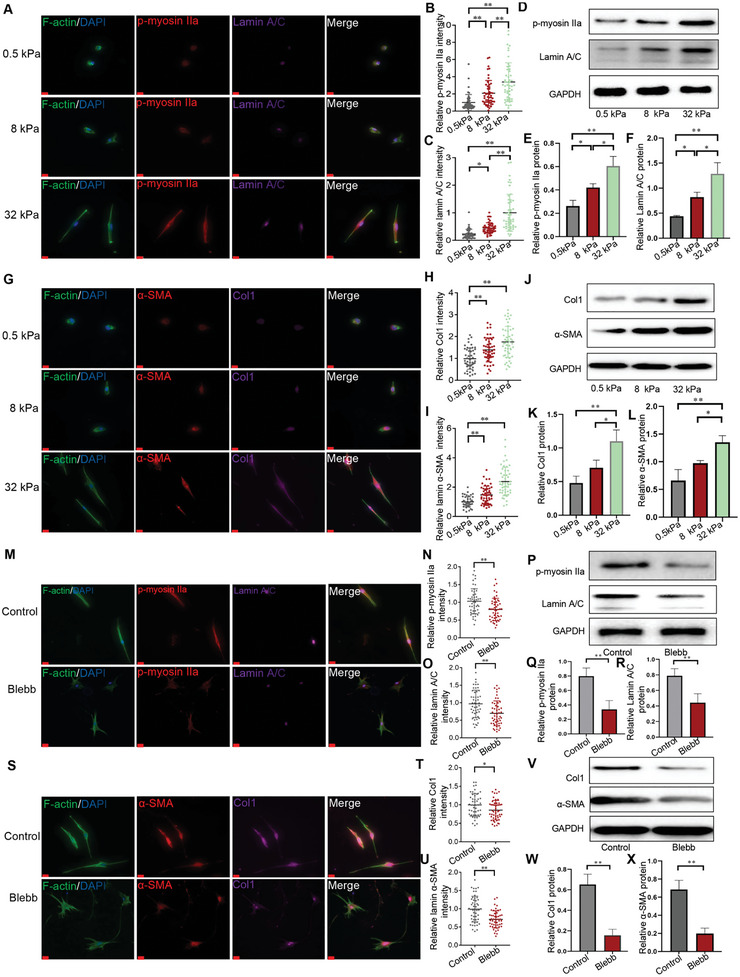
Stiffness regulates bladder FMT via NMIIA‐mediated cellular traction force. A–C) Increased stiffness enhanced the p‐NMIIA and Lamin A/C labeling intensity (*n* = 50). D–F) WB analysis shows the increased stiffness induced the expression of p‐NMIIA and Lamin A/C. G–I) Increased stiffness enhanced the Col1 and α‐SMA labeling intensity (*n* = 50). J–L) WB analysis shows the increased stiffness induced Col1 and α‐SMA expression. M–O) Blebb decreased the p‐NMIIA and Lamin A/C labeling intensity. P–R) WB analysis shows that Blebb decreased the p‐NMIIA and Lamin A/C expression. S–U) Blebb decreased the Col1 and α‐SMA labeling intensity. V–X) WB analysis shows that Blebb decreased the Col1 and α‐SMA expression. Scale bar = 20 µm; *p < 0.05, **p < 0.01; Shown is the mean ± SD.

Bladder fibrosis resulting from BOO is characterized by increased tissue stiffness due to excessive ECM deposition, which in turn leads to an increase in membrane tension felt by cells. The possibilities raise the next question of whether the increased tissue stiffness results in bladder FMT. To investigate this, hydrogels with varying moduli (soft: 0.5 kPa, middle: 8 kPa, stiff: 32 kPa) were used to mimic the stiffness range from normal to fibrotic bladder, according to the elasticity modulus measured in human bladder tissues and previous study.^[^ ^21^ ^]^ It is well known that Lamin A/C, one nuclear structural protein, changes its construction in response to the environmental physical factors.^[^ ^22^ ^]^ With the substrate stiffness increase, there was a significant increase in the p‐NMIIA, Lamin A/C expression as well as cell spread area when cells were cultured 48 h, which was confirmed by both immunofluorescence staining (**Figure** **3**A–C) and WB analysis (Figure 3D–F). Moreover, the increase in protein abundance of Col1 and α‐SMA assessed by WB (Figure 3J–L) and label intensity assessed by immunofluorescence were statistically significant (Figure 3G–I), although the increase was not obvious compared to TGF‐β1 treatment (Figure S2C, Supporting Information). Blebbistatin (Blebb), an NMIIA inhibitor, dramatically decreased the p‐NMIIA and Lamin A/C expression (Figure 3M–R) and exhibited a notable decrease of Col1 and α‐SMA expression both in immunofluorescence analysis (Figure 3S–U) and WB analysis (Figure 3V–X) when fibroblasts cultured on the stiffer substrate(TCPs), indicating that NMIIA was capable of sensing and transducing microenvironmental stiffness signals and the increased stiffness‐induced profibrotic alterations are at least partially dependent on NMIIA activity.

### 2.5 Piezo1 Mediates Stiff ECM‐Induced Bladder FMT

**Figure 4:**
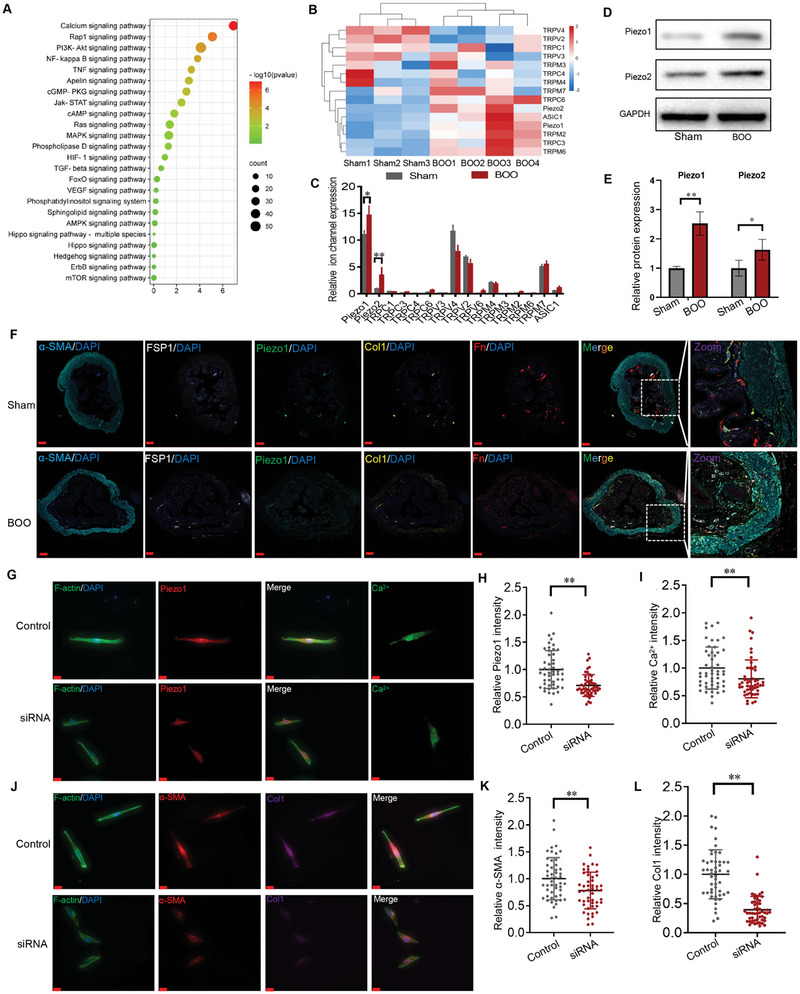
Piezo1 mediates stiff ECM‐induced bladder FMT. A) KEGG enrichment analysis of the DEGs between the sham‐operated mice bladder and BOO mice bladder indicated that the calcium ion pathway was chiefly responsible for BOO‐induced bladder remodeling (*n* = 3). B) Cluster analysis of all ion channels in the transcriptome sequencing results. C) Cluster analysis of all ion channels in the transcriptome sequencing results show that Piezo1 messenger RNA levels were the most abundantly expressed channel and it was prominently upregulated in the BOO bladder. D) Piezo subtypes expression in sham‐operated mice bladder and BOO mice bladder were detected by WB and quantification(blow) shown in (E) (*n* = 3). F) Immunofluorescence was performed to co‐localize cellular traction force markers and activated fibroblast markers (*n* = 3). Scale bar = 200 µm. G–I) Inhibition of Piezo1 by siRNA was confirmed at functional (Ca^2+^ influx) and biochemical (IF) levels; Scale bar = 20 µm. J–L) Inhibition of Piezo1 by siRNA decreased the Col1 and α‐SMA intensity (*n* = 50); Scale bar = 20 µm. *p < 0.05, **p < 0.01;Shown is the mean ± SD.

From the results above, NMIIA‐mediated traction forces are chiefly responsible for cellular mechanotransduction in stiffness‐induced bladder FMT. However, whether NMIIA is the sole stiffness‐sensing mediator and the underlying mechanism of NMIIA‐mediated signal transduction remains unclear. Previous studies have suggested that Ca^2+^ signals play a critical role in promoting fibroblast activation,^[^ ^23^ ^]^ while recent opinions have shown that regulation of calcium influx by ion channels may influence actomyosin contraction and relaxation.^[^ ^24^ ^]^ KEGG enrichment analysis of the DEGs between the sham‐operated mice bladder and BOO mice bladder exhibited that among the signal transduction pathways, the calcium ion pathway was chiefly responsible for BOO‐induced bladder remodeling (**Figure** **4**A). Furthermore, expression analysis from the Human Protein Atlas (HPA) database showed that the bladder had the fourth highest expression of Piezo1, indicating a possible critical role in the bladder (Figure S3, Supporting Information). Cluster analysis of all ion channels in the transcriptome sequencing data indicated that basal Piezo1 messenger RNA level was the most abundantly expressed channel and was prominently upregulated in the BOO bladder compared to the sham‐operated bladder (Figure 4B,C), whose variation trend of Piezo1 was similar to BPH bladder (Figure S4, Supporting Information). WB analysis demonstrated that BOO caused a pronounced increase in Piezo1 and Piezo2 protein expression in bladder tissues (Figure 4D,E). Additionally, multiple staining colocalizing Piezo1 with fibroblast activation markers in fibroblast marked by FSP1 showed that the increased expression of fibroblast activation markers positively correlated with Piezo1 expression, indicating that Piezo1 might have a crucial role in bladder FMT (Figure 4F). To further investigate the special role of Piezo1 in bladder FMT, Piezo1 siRNA was utilized. Piezo1 inhibition by siRNA (Figure 4G–I) attenuated stiffer substrate‐increased protein abundance of Col1 and α‐SMA by immunostaining (Figure 4J–L) and WB analysis (Figure S5, Supporting Information), suggesting that Piezo1 was involved in the stiffer‐induced FMT.

### 2.6 The Crosstalk Between Piezo1 and NMIIA in the Stiffness‐Induced FMT

**Figure 5:**
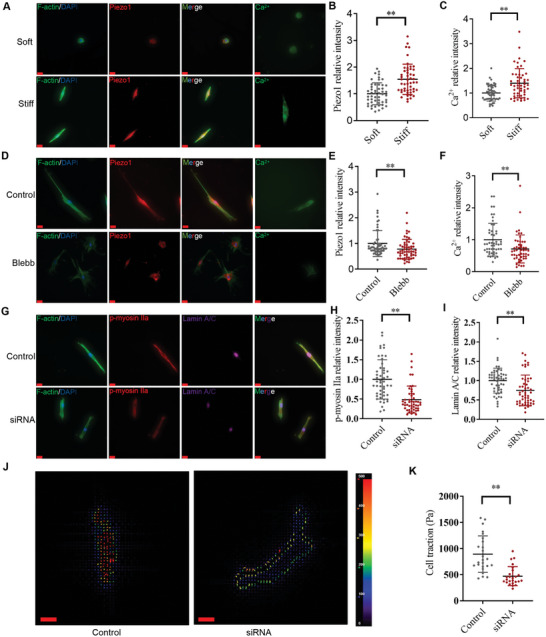
The crosstalk between Piezo1 and NMIIA in the stiffness‐induced FMT. A–C) Stiffen substrate‐activated Piezo1 was confirmed at functional (Ca^2+^ influx) and biochemical (IF) levels. D–F) Blebb decreased Piezo1 was confirmed at functional (Ca^2+^ influx) and biochemical (IF) levels. G–I) Inhibition of Piezo1 by siRNA decreased the p‐NMIIA and Lamin A/C labeling intensity (*n* = 50). J,K) TFM results show that inhibition of Piezo1 decreased the traction forces (*n* = 25). Scale bar = 20 µm; *p < 0.05, **p < 0.01; Shown is the mean ± SD.

The above findings suggest that there are two potential mediators, Piezo1 and NMIIA, involved in stiff‐sensing in bladder FMT. To investigate whether Piezo1 and NMIIA independently mediate stiff‐sensing, cells were cultured on varying stiffness substrates. Similar to TGF‐β1 treatment (Figure S4B, Supporting Information), stiffer substrates not only increased the expression of Piezo1, but also activated the Piezo1 channel, which was confirmed by the increased intracellular Ca^2+^ level when compared to the softer substrate (**Figure** **5**A–C). Inhibition of NMIIA by Blebb (20 µM) attenuated the increased expression of Piezo1 and intracellular Ca^2+^ level of bladder fibroblast induced by stiffer substrates (Figure 5D–F). Additionally, inhibition of Piezo1 by siRNA markedly decreased the expression of Lamin A/C and p‐NMIIA (Figure 5G–I). Furthermore, analysis of TFM results indicated that cellular force was dramatically decreased in the siRNA group (Figure 5J). Taken together, these data support the notion that Piezo1 and NMIIA are involved in a directed interplay in the physical stiffness mechanotransduction process.

**Figure 6:**
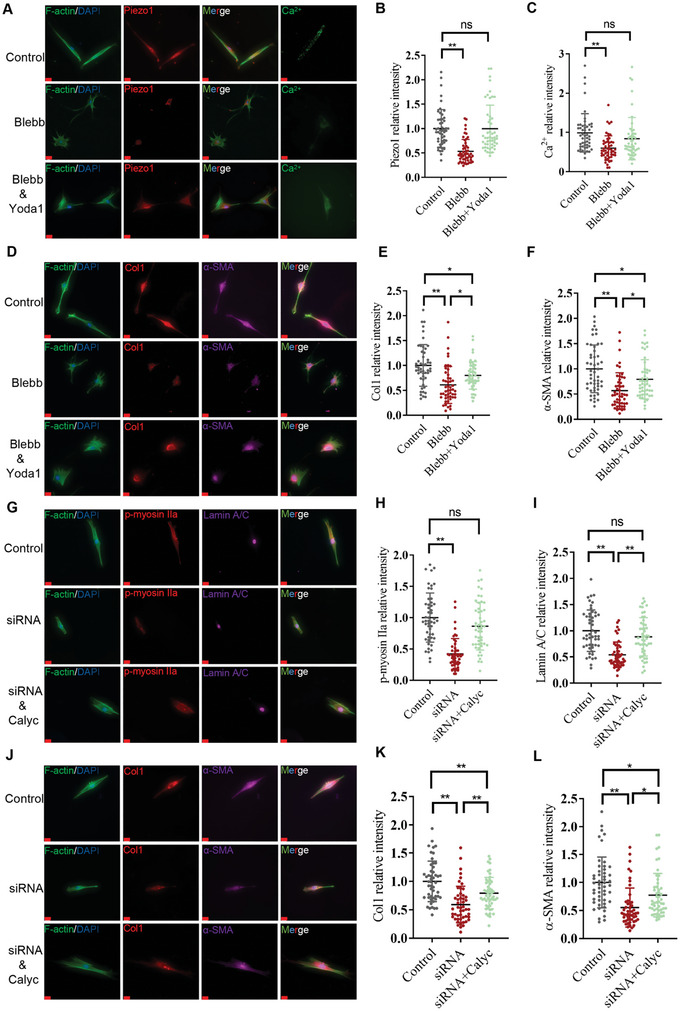
Piezo1 and NMIIA could directly and indirectly influence the stiffness‐induced FMT. A–C) After the treatment with Blebb, concertation of Yoda1 to adjust the intracellular Ca^2+^ to a baseline level as the control group. D–F) Yoda1 attenuated the Blebb‐decreased fibroblast activated markers, while still lower than the control group (*n* = 50). G–I) Concertation of Calyc to adjust the p‐NMIIA expression to baseline level of the control group (*n* = 50). J–L) Blebb attenuated the siRNA Piezo1‐decreased fibroblast activation markers, while still lower than the control group (*n* = 50). Scale bar = 20 µm; *p < 0.05, **p < 0.01; n.s, not significant. The data are expressed as the mean±SD of three independent experiments.

The above findings suggest that the activity of Piezo1 and NMIIA in bladder FMT may be interdependent, but their causal relationship remains unclear. To investigate whether NMIIA‐mediated fibroblast activation depends on Piezo1 activity, we added Yoda1(0.1 nm), a Piezo1 agonist, following the Blebb treatment to revert back the intracellular Ca^2+^ levels to their pre‐treatment state (**Figure** **6**A–C). However, Yoda1 treatment attenuated, but did not completely abolish, the Blebb‐induced decrease in Col1 and α‐SMA expression (Figure 6D–F). These data suggest that stiff‐sensing via the NMIIA pathway also occurs through an alternative pathway independent of NMIIA/Piezo1. Likewise, Piezo1 knockdown predominantly decreased the fibroblast activation markers accompanied by a decrease in NMIIA. To investigate whether Piezo1‐mediated fibroblast activation depends on NMIIA activity, we added Calyculin A (Calyc) (0.1 nm), an NMIIA agonist, following siRNA Piezo1 treatment to revert back the p‐NMIIA expression to the basal level of the control group (Figure 6G–I). Blebb treatment suppressed, but failed to abolish the knock down Piezo1‐induced decrease in Col1 and α‐SMA expression (Figure 6J–K). These data suggest that stiff‐sensing via Piezo1 activation also occurs through an alternative pathway independent of Piezo1/NMIIA. Collectively, Piezo1‐mediated Ca^2+^ and NMIIA‐mediated traction force have a complex feedback circuit in stiffness‐induced bladder FMT, and both pathways may involve other unknown pathways in addition to affecting each other.

### 2.7 Myosin II Activated Piezo1 Through PI3K/PIP3 Mediated Membrane Tension

**Figure 7:**
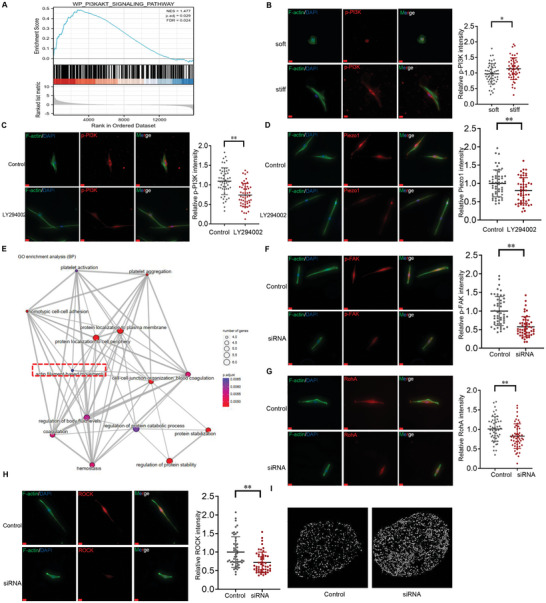
The underlying mechanism of the crosstalk between *Myosin II and Piezo1*. A) GSEA data analysis shows PI3K and focal adhesion pathway were significantly altered in BOO bladder. B) Stiffen substrate activated p‐PI3K labeling intensity (*n* = 50). C) PI3K inhibitor LY29004 decreased the p‐PI3K labeling intensity (*n* = 50). D) LY294002 decreased the Piezo1 labeling intensity (*n* = 50). E) Co‐immunoprecipitation coupled to mass spectrometry assay enriched in actin‐filament and plasma membrane; F) Inhibition of Piezo1 by siRNA decreased the p‐FAK labeling intensity (*n* = 50). G) Inhibition of Piezo1 by siRNA decreased the RhoA labeling intensity (*n* = 50). H) Inhibition of Piezo1 by siRNA decreased the ROCK labeling intensity. I) Inhibition of Piezo1 by siRNA results in condensation of chromatin (*n* = 15, two technical replicates), Scale bar = 20 µm; *p < 0.05, **p < 0.01; Shown is the mean ± SD.

We next investigate the molecular mechanisms underlying the crosstalk between Piezo1 and NMIIA in bladder FMT. Piezo1 is a transmembrane protein that responds to mechanical force by transiently opening and increasing plasma membrane tension.^[^ ^25^ ^]^ The current view holds that the phosphoinositide turnover of PIP2 (phosphatidylinositol‐4,5‐bisphosphate) and PIP3 play a critical role in controlling membrane tension.^[^ ^26^, ^27^ ^]^ PI3K serves as an activator facilitating the breakdown of PIP2 into PIP3 representing increase the membrane tension.^[^ ^28^ ^]^ PI3K‐mediated PIP2/PIP3 signaling is therefore potentially associated with Piezo1 activation. The GSEA data from the mice bladder indicated that the PI3K pathway was primarily involved in remodeling (**Figure** **7**A). To further explore the role of membrane tension in Piezo1 activation induced by stiffness, cells were cultured on soft (0.5 kPa) and stiff (32 kPa) substrates for 48 hours, and immunofluorescence results showed that the stiff substrate caused a significant increase in the expression of p‐PI3K and Piezo1 (Figure 7B). LY29004 (10 uM), a PI3K inhibitor, significantly decreased the labeling intensity of p‐PI3K as well as Peizo1 (Figure 7C,D). These findings suggest that the activation of Piezo1 by NMIIA‐generated force is likely regulated by the PI3K‐mediated PIP2/PIP3 pathway.

### 2.8 Piezo1 Mediated Ca2+ Activated Myosin II Through FAK/RhoA/ROCK Pathway

We investigated how Piezo1 regulates NMIIA by performing immunoprecipitation of Piezo1 in fibroblasts from both soft and stiff substrates. The top 50 DEGs from the immunoprecipitation coupled to mass spectrometry assay were enriched in actin filaments and plasma membrane, indicating Piezo1's likely involvement in cellular traction force (Figure 7E). The canonical signaling pathway responsible for stiffness mechanotransduction is FAK/RhoA/ROCK.^[^ ^29^, ^30^ ^]^ To investigate whether Piezo1‐mediated Ca^2+^ influx regulates NMIIA via this pathway, we measured the labeling intensity of p‐FAK, RhoA, and ROCK after treating the cells with siRNA Piezo1. The labeling intensity of all three markers significantly decreased after the siRNA Piezo1 treatment (Figure 7F–H), suggesting that Piezo1‐mediated NMIIA likely occurs via the FAK/RhoA/ROCK pathway on a stiff substrate. Additionally, the chromatin condensation parameter (CCP) results supported our conclusion that chromatin is more condensed in nuclei after siRNA Piezo1 treatment (Figure 7I).

### 2.9 Genetic Deletion of Piezo1 in Fibroblast Attenuates BOO Remodeling and Bladder Dysfunction

**Figure 8:**
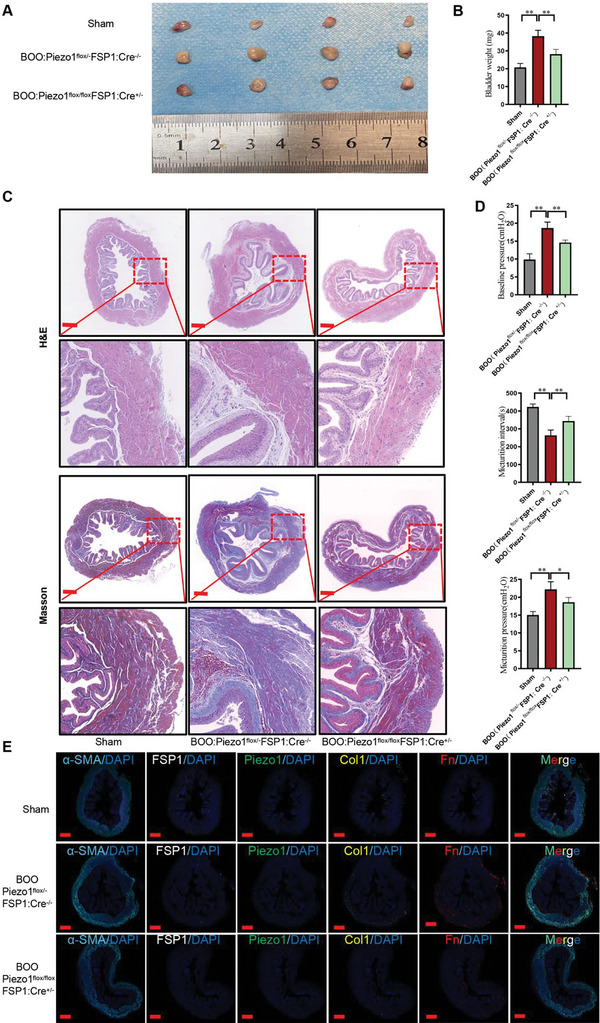
Deletion of fibroblast Piezo1 attenuates BOO remodeling and bladder dysfunction. A) Bladder harvested from sham‐operated, BOO, and deletion of fibroblast in BOO group (*n* = 4). B) Bladder weight in sham‐operated, BOO, and deletion of fibroblast in BOO group (*n* = 4). C) HE and Masson staining of bladder in sham‐operated, BOO, and deletion of fibroblast in BOO group (*n* = 3). D) Urodynamic parameters analysis in sham‐operated, BOO, and deletion of fibroblast in BOO group (*n* = 3). E) Immunofluorescence was performed to co‐localize Piezo1 and activated fibroblast markers of bladder in sham‐operated, BOO, and deletion of fibroblast in BOO group (*n* = 3); *p < 0.05, **p < 0.01. Shown is the mean ± SD.

BOO‐induced fibrotic bladder is characterized by an increase in collagen expression and cell proliferation. Therefore, treatments targeting BOO mainly focus on inhibiting collagen synthesis and cell proliferation. The present study demonstrates that Piezo1, in addition to its role in bladder FMT, also regulates fibroblast proliferation, as evidenced by PCNA expression and EdU analysis. To investigate the specific role of Piezo1 in bladder remodeling induced by BOO, we generated an inducible fibroblast‐specific Piezo1 conditional knockout mouse model. Tamoxifen‐inducible Cre‐FSP1 transgenic mice were crossed with Piezo1^flox/flox^ mice, and offspring backcrossed until Piezo1^flox/flox^FSP1^+/−^ mice were generated (Figure S7, Supporting Information). The results showed that bladder weight significantly increased in the BOO group compared to the sham‐operated group, while deletion of Piezo1 in bladder fibroblasts markedly attenuated it (**Figure** **8**A,B). Masson and HE staining showed that the BOO exhibited thickened muscle layers and an increased accumulation of ECM, which was reversed by the deletion of Piezo1 in bladder fibroblast (Figure 8C). Additionally, deletion of fibroblast Piezo1 suppressed the BOO‐decreased micturition interval as well as the enhanced micturition pressure, baseline pressure, and threshold pressure (Figure 8D). To emphasize Piezo1's distinctive role in cell proliferation and collagen synthesis, we conducted an immunofluorescence analysis of α‐SMA, PCNA, and Col1. Our observations revealed that Piezo1 expression, along with these markers, increased in the BOO group, which was reversed by the deletion of Piezo1 in bladder fibroblast (Figure 8E). To further validate the feasibility of drug intervention, we applied the Piezo1 inhibitor GsMTx4, and found that GsMTx4 may significantly alleviate bladder fibrosis induced by BOO (Figure S8, Supporting Information). Hence, the in vivo experiment initially confirms the relationship between Piezo1 and stiffness‐induced bladder fibrosis.

## 3 Discussion

Emerging studies indicate a positive correlation between tissue stiffness and the development of fibrotic conditions in various organs such as the lung, liver, and kidney.^[^ ^13^, ^31^, ^32^ ^]^ BOO‐induced bladder fibrosis causes the bladder tissue much stiffer (≈32 kPa) than normal bladder tissue (≈0.5 kPa), which creates an aberrant mechanical microenvironmental stimulus to bladder cells.^[^ ^21^ ^]^ Previous studies indicated bladder smooth muscle cells, urothelial cells, and macrophages were all involved in the ECM components deposition including Col1, Col2, Col3, and FN1,^[^ ^3^ ^]^ while few know about the fibroblasts. The present study demonstrates that bladder fibroblasts, which secrete the most collagens among all bladder cells, are primarily involved in ECM remodeling. FMT as well as fibroblast activation, accompanied by increased α‐SMA expression and ECM components, is predominantly involved in the fibrotic condition, which remains unclear in the bladder remodeling. Therefore, clarifying the underlying mechanism of mechanotransduction in FMT secondary to BOO offers a high potential for developing effective therapeutic approaches for early clinical intervention.

Stretch‐activated ion channels, which are triggered by mechanical stimuli such as touch, membrane tension, traction force, and shear stress, permit the passage of ions.^[^ ^33^ ^]^ Piezo1 is a newly discovered stretch‐activated ion channel that has been implicated in a range of physiological and pathophysiological processes, including proliferation, fibrosis, and innate immunity in various cell types.^[^ ^24^ ^]^ We found that the DEGs from RNA sequencing between mice with BOO and sham‐operated bladder enriched into the calcium channel pathway, with Piezo1 being the highest basal expression calcium channel and significantly increased in BOO bladder. This finding suggests that Piezo1 may be activated and up‐regulated when fibroblasts respond to the stiffen micro‐environment induced by BOO. Consistent with the previous studies,^[^ ^34^ ^]^ our findings indicate that stiff substrate‐induced FMT dependent on NMIIA. On the other hand, NMIIA is the dominant generator of traction forces for probing the stiffness of the extracellular matrix, and is usually considered an evaluation index for the traction force.^[^ ^35^ ^]^ In this work, NMIIA expression was significantly increased in both human and mouse fibrotic bladder specimens when compared to normal controls, particularly in fibroblast. One potential explanation for these findings was due to the increased stiffness induced by ECM deposition secondary to BOO which is dependent on NMIIA activity.

Although the crucial role of the two mechanosensors, Piezo1 and NMIIA, in stiffness‐induced fibroblast activation is well established, their interplay in fibroblast response to substrate stiffness and whether they have a synergistical effect remains unclear. Our findings show that Piezo1‐mediated Ca^2+^ influx may influence the activity of NMIIA and intracellular force. Alternatively, NMIIA‐generated intracellular forces influence the Piezo1 expression and activity, which is consistent with the notion in other groups.^[^ ^36^ ^]^ These observations indicated Piezo1 and NMIIA have a direct physiological crosstalk in the mechanotransduction process. Inhibition of Piezo1‐ and NMIIA‐ independently fail to revert back the stiff‐increased fibroblast activation markers expression to the basal level of the control group indicating the stiff‐sensing through an alternative pathway independent of Piezo1/NMIIA pathway, respectively. These findings demonstrate that Piezo1 and NMIIA are integrated through a complex feedback circuit, synergistically resulting in stiffness‐induced bladder FMT.

Piezo1 is a plasma membrane protein, and we hypothesize that plasma membrane tension is sufficient to regulate the open probability of Piezo1.^[^ ^37^ ^]^ Previous evidence has suggested that PIP2/PIP3 pathway, which is regulated by PI3K, is closely linked to membrane tension, and the PI3K activity indirectly reflects the degree of membrane tension^[^ ^26^, ^27^ ^]^ We demonstrated the NMIIA‐generated intracellular force activates Piezo1, possibly via PI3K/PIP3 pathway. Alternatively, Piezo1 may regulate NMIIA activity and NMIIA‐based traction forces. Several signaling pathways have been reported to modulate NMIIA activity, among which the FAK/RhoA/ROCK pathway has been extensively studied.^[^ ^38^ ^]^ The remodeling of focal adhesion complexes connected to the cytoskeleton via the NMIIA‐coupled actin stress fibers contributes significantly to the mechanical responsiveness of stiffness. We demonstrated Piezo1‐regulated Ca^2+^ influx may enhance intracellular force by classical FAK/RhoA/ROCK pathway. The potentially robust feed‐forward loop between Piezo1 and NMIIA intrigues us to demonstrate knockdown Piezo1 increased intensity clusters by CCP analysis, which is consequently related to the cell fate. ECM accumulation and cell proliferation is the primarily character of the bladder fibrosis secondary to BOO. Besides the critical role in FMT, which significantly enhanced the ECM accumulation, we reveal that inhibition Piezo1 suppresses the proliferation. Together, the combined feedback mechanisms provide a strategy for the therapy of bladder fibrosis.

**Figure 9:**
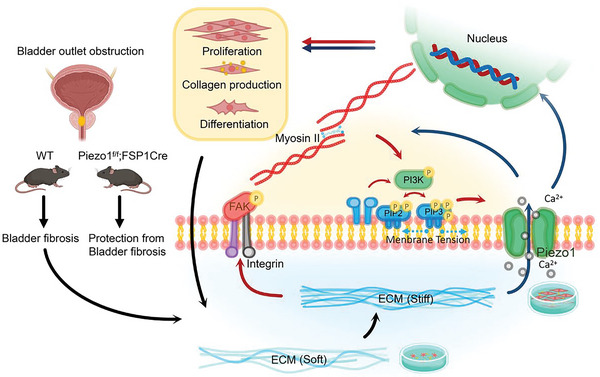
Schematic illustration of mechanotransduction circuit in fibroblasts responding to stiffness: Myosin II and Piezo1 have a feed‐forward crosstalk through the PI3K/PIP3 pathway‐mediated membrane tension and the FAK/RhoA/ROCK pathway, which synergistically contribute to stiffness‐induced fibroblast activation.

In light of the significant and wide‐ranging impact of bladder fibrosis and the availability of Piezo1‐floxed mice, we were driven to investigate the effects of fibroblast‐specific deletion of Piezo1 in BOO mice. These findings highlight the potential of Piezo1 as a therapeutic target for the management of bladder fibrosis and associated bladder dysfunction (**Figure** **9**). GsMTx4, a Piezo1 inhibitor, demonstrated a similar beneficial effect supporting the results.

## 4 Conclusion

Here we investigated the role and mechanism of NMIIA‐based traction forces and Piezo1‐mediated Ca^2+^ influx in regulating bladder FMT response to varying stiffness environments. Above all, we show the positive feedback loop between Piezo1 and NMIIA and synergistically drive FMT response to stiffness. Finally, we target Piezo1 during bladder fibrosis to highlight regulating Piezo1 is likely a novel therapeutic strategy for ameliorating bladder fibrosis and dysfunction.

## 5 Experimental Section

### Mice

C57BL/6J male mice aged 8–12 weeks were used to investigate bladder remodeling during BOO. The BOO surgery was performed as previously described^[^ ^39^ ^]^ and were sacrificed on days 7, 14, and 21 after the obstruction. Piezo1*^flox/flox^* and *S100a4‐Cre*ERT2 mice from Jackson Laboratory (029213) and Gempharmatechn Co. (T006589), respectively, were used to generate the Piezo1*^flox/flox^ S100a4‐Cre*ERT2 mice, which induced the specific fibroblast knockout of the Piezo1 gene by tamoxifen administration (2 mg per day intraperitoneally for 5 days), then age‐ and gender‐matched mice were anesthetized for the establishment of the BOO. Additionally, BOO were intraperitoneally injected with 10 mg kg^−1^ GsMTx4 every other day, starting on the first day after the surgery to evaluate the role of Piezo1 in the BOO‐induced fibrosis. The animal experiments were in adherence with the National Institutes of Health Guidelines and approved by the West China Forth Hospital Committee on Animal Care (approval Gwll2023006).

### Human Tissue Specimens

Five surrounding bladder tissues of bladder tumor and bladder tissues of BPH were harvested for Single‐cell RNA sequencing(scRNA‐Seq) and immunostaining. This study was approved by the Medical Ethics Committee of West China Hospital, Sichuan University (approval 20201282).

### Cell and Reagents

Normal primary human bladder stromal fibroblasts (HBdSFs, No. 4330, ScienCell, San Diego, CA) were cultured with special Fibroblast Medium (FM, Cat. #2301) containing 1% FBS and 1% penicillin/streptomycin. Cells between passages 2 and 6 were incubated in conventional culture conditions (5%CO_2_ and 37 °C) for all experiments. HBdSFs were treated with the indicated intervention reagents after exposure to hydrogels with different stiffness and TCPs for 2 days. The intervention reagents included TGF‐β1 (10 ng mL^−1^, R&D Systems, 7754‐BH), GsMTx4 (1 µm, TAIJIA biotech, TJ8434), Yoda1 (0.1 µm, MedChemExpress, HY‐18723), Calyculin A (Calyc, 0.1 nm, Abcam, ab141784), (‐)‐Blebbistatin (20 µm, MERCK, B0560), and LY29004(0.1 nm, Abcam, ab120243).

### Polyethylene Glycol (PEG)‐RGD Hydrogel

Diacrylate‐terminated PEG (CAS NO.26570‐48‐9, Sigma) reacted with cyclo(RGDfc) to perform PEG‐RGD hydrogels in 2 cm^2^ and 9.6 cm^2^ plates with stiffness substrates of 0.5, 8, and 32 kPa (Table S1, Supporting Information) to mimic the stiffness of natural and fibrotic bladder wall, respectively, based on nanoindentation experiments analysis of bladder tissue sections and previously published data.^[^ ^21^ ^]^ Cells were cultured for 6 h, followed by the treatments for another 2 days and then collected for protein analysis. The proliferation was detected by using an EdU labeling kit (RIBOBIO, C10310‐1) according to the manufacturer's instruction. Fluorescence images were obtained by fluorescence microscopy (Leica). The proportion of EdU‐staining positive cells to the total cells labeled by Hoechst indicated the proliferative rate.

### Nanoindentation

Nanoindentation experiments were performed using the Chiaro system (Optics11, Amsterdam, Netherlands). Optical and geometrical calibrations were performed according to the manufacturer's instructions to test tissue stiffness with one 20 µm‐thick slice at the center of the human or mouse bladder tissues.

### Cell Transfection

HBdSFs cultured on hydrogels and TCPs were transfected with 100 nm siRNAs by using Lipofectamine RNAi MAX reagent according to the manufacturer's protocol. At 48 h after transfection, cell protein expression in siRNA‐treated was assessed by Western blotting. The sequences of control and Piezo1 siRNAs are listed in Table S2 (Supporting Information).

### Fluo‐4 Single Cell Ca^2+^ Imaging

After the treatment, the HBdSFs were loaded with Fluo‐4‐AM (1 mm; Invitrogen, Cambridge, UK) for 1 h. Then, the HBdSFs were washed with phosphate‐buffered saline (PBS) 3 times and then rested for 20 min to allow complete processing of the AM ester by intracellular esterases. Fluorescence images of Ca^2+^ were taken with fluorescence microscopy (Leica).

### Western Blot (WB) Analysis

The procedures of WB were as previously described.^[^ ^39^ ^]^ GAPDH band density was used as a loading control. The membranes were incubated overnight at 4 °C with the following antibodies: GAPDH (ab9485; 1:2500 dilution; 37 kDa), Col1 (ab96723; 1:1000 dilution; 129 kDa), Fn (ab268020; 1: 1000 dilution; 262 kDa), and PCNA (ab29; 1:1000 dilution; 29 kDa) from Abcam, α‐SMA (48938; 1:1000 dilution; 42 kDa) from Cell Signaling Technologies, Piezo2 (NBP1‐78624; 1:1000 dilution; 82 kDa) and Lamin A/C (NB100‐74451; 1:1000 dilution; 70 and 65 kDa) from NOVUS; Piezo1 (APC087; 1:1000 dilution; 257 kDa) from (Alomone Laboratories, Israel) followed by HRP‐tagged secondary antibodies (1:2000 dilution) for 1 h at room temperature. The membranes were then exposed to the ECL chemiluminescence kit, and the integrated density of the bands was quantified using Image J software (NIH, USA).

### Immunofluorescence (IF) Staining

Bladder specimens and cells were fixed, permeabilized, and labeled for filamentous actin (F‐actin) (2219253, 1:500 dilution) from Invitrogen; Piezo1 (APC087, 1:200 dilution) from Alomone Laboratories, p‐FAK (ab81298, 1:200 dilution), RhoA (ab187027, 1:200 dilution), ROCK (ab97592, 1:200 dilution), and Col1 (ab138492, 1:200 dilution) from Abcam; α‐SMA (48938, 1:200 dilution), p‐PI3K (17366, 1:200 dilution) and p‐Myosin (14611S, 1:200 dilution) from Cell Signaling Technologies; Lamin A/C (NB100‐74451, 1:200 dilution) from NOVUS at 4 °C overnight. Then washed with PBS and incubated with secondary antibodies anti‐Rabbit Alexa Fluor 568 (Thermo Scientifific, A‐11011, 1:500 dilution) and Goat anti‐Mouse Alexa Fluor 647 (Thermo Scientifific, A‐32728, 1:500 dilution) for 1 h at room temperature as the previous study.^[^ ^40^ ^]^ Immunofluorescence intensity was analyzed using Image J Pro software (NIH). Greater than 50 cells for each condition were analyzed in triplicate.

### Traction Force Microscopy (TFM)

Hydrogels containing 0.5 µm fluorescent carboxylated polystyrene beads (latex beads, carboxylate‐modified polystyrene, fluorescent green, Sigma) were fabricated as previously described.^[^ ^40^ ^]^ The Fiji Image J plugin “Align slices in stack” was used to correct the experimental drift of the samples. The displacement field in a spread cell region was subsequently calculated by a “particle image velocimetry” plugin in Image J. The obtained result was reconstructed using the “Fourier transform traction cytometry” plugin to generate the traction force field as a vector plot.

### Chromatin Condensation Parameter (CCP)

To generate chromatin condensation parameters (CCP), the gradient‐based Sobel edge detection algorithm was performed to measure the edge density of individual nuclei by MATLAB script.^[^ ^41^ ^]^

### Cystometry

Mice were anesthetized with isoflurane/O_2_ (3% induction and 1% maintenance) after the treatment. Cystometry was performed to evaluate the urodynamic parameters as previously described.^[^ ^42^ ^]^ The bladder was exposed via a midline abdominal incision and the ligature around the urethra for BOO surgery was removed. SP 31 catheter (inside diameter: 0.5 mm, Natsume, Tokyo, Japan) was inserted into the bladder dome and secured. Then, the abdominal incision was closed. Saline was infused into the bladder (0.6 mL h^−1^) and mice were given a period for the voiding pattern to get stabilized. Thereafter, cystometry was performed to evaluate the urodynamic parameters as previously described. After cystometric studies, bladder weights were measured.

### Immunoprecipitation Followed by Mass Spectrometry (IP‐MS)

For both soft and stiff substrates, cells were harvested in culture media and subjected to protein extraction as described in the manual instruction. The extracted protein samples were subjected to immunoprecipitation using α‐HA magnetic beads (Thermo Fisher Scientific, USA) (50% slurry) overnight at 4 °C with gentle inversion. After washing, the binding protein complex was eluted by 0.1 m glycine (pH 2.0), and half of the eluted samples were preprocessed and analyzed on an LTQ‐Orbitrap Fusion Mass Spectrometer (Thermo Fisher Scientific, USA) coupled with an EASY‐nLC 1000 Liquid Chromatograph (Thermo Fisher Scientific, USA).

The original mass spectrometric data were analyzed using bioinformatics and statistical methods by Beijing Genomics institution. A total of 27199 spectra were obtained from the soft substrate, and after identification by the search engine, 2345 spectra were matched, resulting in the identification of 581 proteins and 1914 peptides. Similarly, a total of 26656 spectra were obtained from the stiff substrate, and after identification by the search engine, 1744 spectra were matched, resulting in the identification of 472 proteins and 1454 peptides. Gene ontology analysis was performed using the top 50 differential expressed genes.

### Statistical Analysis

Each experiment was methodically replicated two or three times with two or three biological replicates. *n* = 50 represents the sample size taken from 2–3 parallel specimens in a single experiment, yet the repeated experiments exhibit the same trend. The data are shown as the mean ± standard deviation (SD) and Group differences were conducted by one‐way ANOVA with Tukey's post ‐hoc test. P‐values < 0.05 were considered as statistically significant (*p < 0.05, **p < 0.01) using GraphPad Prism 7.03 (Graph‐Pad, San Diego, CA).

## Conflict of Interest

The authors declare no conflict of interest.

## Supporting information

Supporting Information

## Data Availability

The data that support the findings of this study are available on request from the corresponding author. The data are not publicly available due to privacy or ethical restrictions.
