# Supplementary material for: Actomyosin Activity and Piezo1 Activity Synergistically Drive Urinary System Fibroblast Activation
Source: Adv Sci (Weinh). 2023 Oct 22;10(33):2303369. doi: 10.1002/advs.202303369 (PMC10667826; doi:10.1002/advs.202303369)
Supplement: Supplementary file 1 — Supporting Information [file ADVS-10-2303369-s001.pdf]

ADVANCED SCIENCE
Open Access

Supporting Information

for *Adv. Sci.*, DOI 10.1002/advs.202303369

# Actomyosin Activity and Piezo1 Activity Synergistically Drive Urinary System Fibroblast Activation

*Guo Chen, Xiaoshuai Gao, Jiawei Chen, Liao Peng, Shuang Chen, Cai Tang, Yi Dai, Qiang Wei* and Deyi Luo**

# Supporting Information

**Table S1.** List of preparation for the PEG-RGD hydrogels with vary stiffness. Concentration of 40 mg/mL, 100 mg/mL, and 200 mg/mL represent 0.5 kPa, 8 kPa, and 32 kPa, respectively.

| Concentration[mg/mL] | Polymer from stock [a] [uL] | Initiator [b] [uL] | PBS [uL] | RGD [c] [uL] |
|---|---|---|---|---|
| 40 | 40 | 4.56 | 405.44 | 50 |
| 100 | 100 | 11.4 | 338.6 | 50 |
| 200 | 200 | 22.8 | 227.25 | 50 |

[a]Stock is PEG700 at 500mg/mL concentration; [b] Initiator( with 70% ethanol);
[c] cyclo(RGDfc was in the stock concentration of 1 mmol/L.

**Table S2.** Sequence of primers for control and Piezo1 siRNAs.

| Target gene | Primer sequence |
|---|---|
| Negative control sense(5'-3') | UUCUCCGAACGUGUCACGUTT |
| Negative control antisense(5'-3') | ACG UGACACGUUCGGAGAATT |
| Piezo1-homo-4155 sense(5'-3') | GCGUCUUCCUUAGCCAUUATT |
| Piezo1-homo-4155 antisense(5'-3') | UAAUGGCUAAGGAAGACGCTT |

**Table S3.** List of the canonical marker genes for ten cell types of the bladder.

| Cell | Canonical marker |
|---|---|
| T cells | CD8A,CD3E,CD3D |
| urothelial cells | UPK1A,UPK3A |
| natural killer cell | NKG7,GNLY |
| plasmocyte | JCHAIN,XBP1,MZB1 |
| myeloid cells | FCGR3B,ADGRG3,CXCR2,S100A8 |
| interstitial cells | KRT13,FSTL4 |
| fibroblasts | DCN,LUM,ACTA2 |
| macrophages | CD86,C1QA,FCER1G |
| endothelials | EPCAM,CLDN3 |
| B cells | CD19,MS4A1 |

**Table S4.** List of the canonically cellular force-related markers expression in BPH bladder and normal bladder by the enrichment analysis of DEGs. FAK(*PTK2*), NMIIA(*MYH9*), ROCK(*ROCK*), etc, were increased in BPH bladder when compared with normal bladder.*, $p < 0.05$.

| Gene | Normal bladder | BPH bladder |
|---|---|---|
| PTK2 | 0.802530428 | 1.195336247* |
| TLN1 | 1.174495498 | 1.084525975 |
| TLN2 | 0.141817583 | 0.083075075 |
| YAP1 | 0.433582787 | 0.364782807 |
| ROCK1 | 1.879217206 | 2.388932668* |
| ROCK2 | 0.781891777 | 0.93754105 |
| PIK3CA | 0.544937551 | 0.743781832* |
| PIK3CB | 0.555071315 | 0.656584913* |
| PIK3CD | 0.561614033 | 0.785800739* |
| PIK3R1 | 2.567934991 | 2.851300097 |
| PIK3R2 | 0.056637427 | 0.064262481* |
| PIK3R3 | 0.256319246 | 0.288205671 |
| PTEN | 1.22335159 | 1.321430277 |
| PXN | 0.26966839 | 0.217634509 |
| MYH9 | 2.712477962 | 3.145923604* |
| MYH10 | 0.12385851 | 0.142577952 |
| Flna | 0.990069885 | 0.803998578 |
| Flnb | 0.483142804 | 0.524825885 |
| Flnc | 0.017284545 | 0.004642426 |

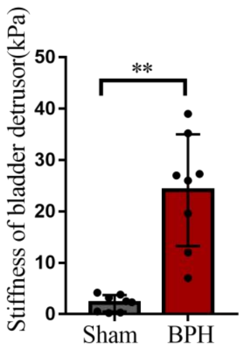


**Figure S1.** The elasticity modulus of the BPH bladder wall was proudly increased compared with the normal bladder ($n = 8$). **, $p < 0.01$. The data are expressed as the mean ± SD of three independent experiments.

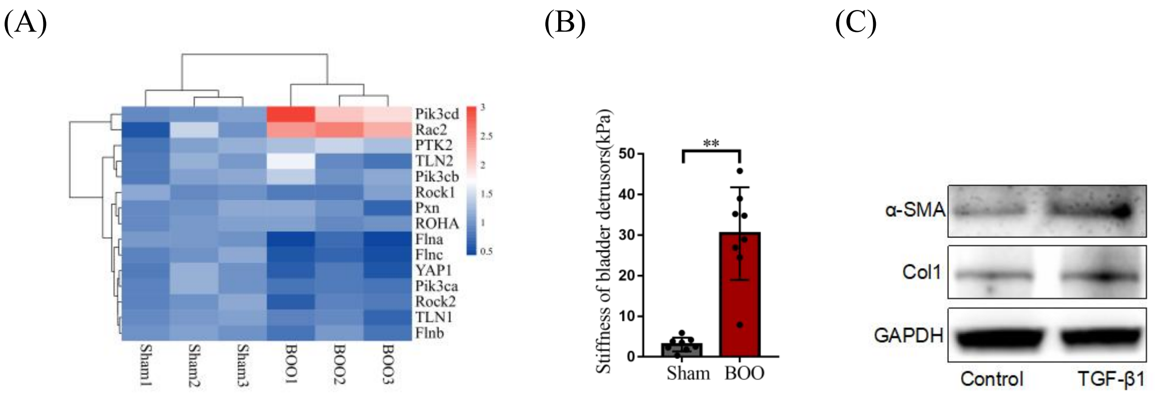


**Figure S2.** (A). Microarray analysis of cell traction force-related genes indicated that markers such as PI3K, Rac, etc. were significantly increased in BOO bladder. (B). Elasticity modulus of the BOO bladder wall was proudly increased compared with the sham-operated group ($n = 4$, two biological replicates). (C). TGF-β1 treatment proudly increase protein abundance of Col1 and α-SMA by WB analysis ($n = 3$). **, p<0.01.The data are expressed as the mean ± SD

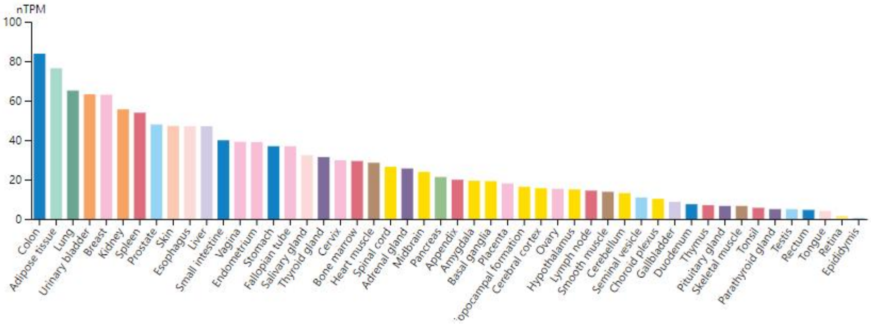


**Figure S3.** Expression analysis from the HPA database(consensus dataset) showed that bladder was the organ with fourth high expression of Piezo1.

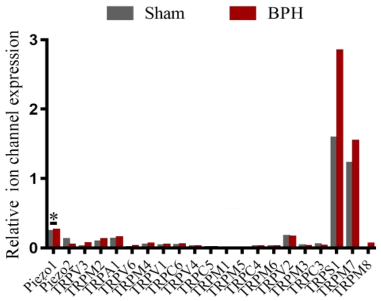


**Figure S4.** Piezo1 messenger RNA level was prominently upregulated in the BPH bladder when compared with those in the Sham(normal) bladder ($n = 5$), * , $p < 0.05$.

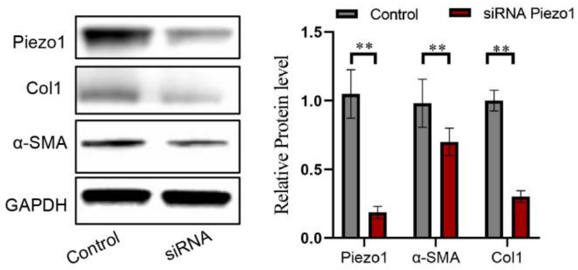


**Figure S5.** Piezo1 inhibition by siRNA attenuated stiffer substrate-increased protein abundance of Col1 and α-SMA by WB analysis ($n = 3$). **, $p < 0.01$.The data are expressed as the mean ± SD of three independent experiments.

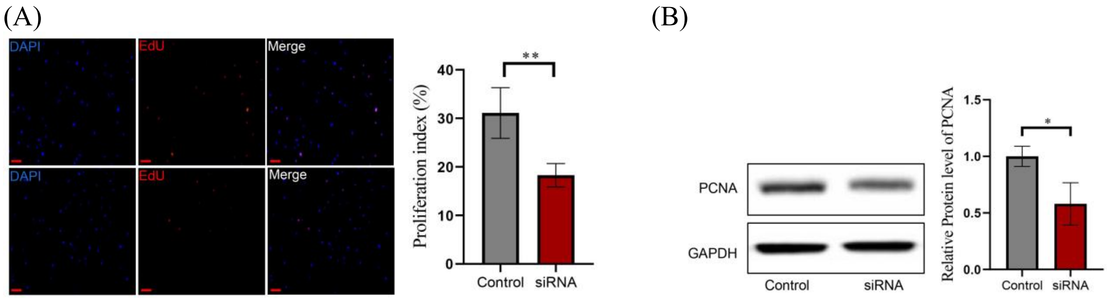


**Figure S6.** (A). Inhibition Piezo1 by siRNA treatment suppressed fibroblast proliferation by EDU analysis. Scale bar = 100 µm. (B) Piezo1 by siRNA treatment suppressed fibroblast proliferation, which was confirmed by the PCNA expression ($n = 3$). **, $p < 0.01$. The data are expressed as the mean ± SD of three independent experiments.

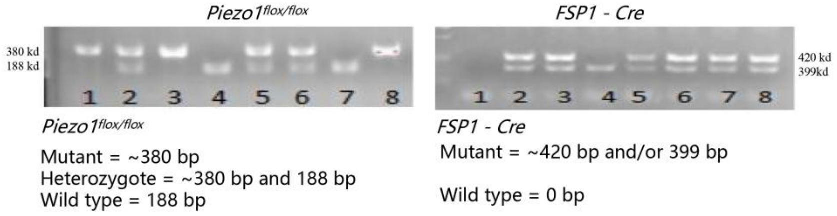


**Figure S7.** Tamoxifen-inducible Cre-FSP1 transgenic mice were crossed with Piezo1$^{flox/flox}$ mice, and offspring backcrossed until Piezo1$^{flox/flox}$FSP1$^{+/-}$ mice were generated (3 and 8).

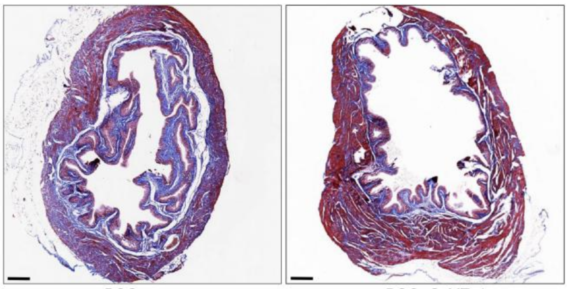


**Figure S8.** GsMTx4(10ug/g) significantly alleviate bladder fibrosis induced by BOO; scale bar = 200 µm.
